# Application of comprehensive molecular genetic profiling in precision cancer medicine, Hungarian experiences

**DOI:** 10.2340/1651-226X.2024.39918

**Published:** 2024-06-16

**Authors:** Erika Tóth, Zsófia Küronya, Edina Soós, Tamás Pintér, Henriett Butz, Zsolt Horváth, Erzsébet Csernák, Vince Kornél Grolmusz, Judit Székely, Tamás Strausz, József Lövey, Levente Jánvári, László Báthory-Fülöp, Péter Nagy, Csaba Polgár, Attila Patócs

**Affiliations:** a National Institute of Oncology, National Tumor Biology Laboratory, Budapest, Hungary; b Department of Surgical and Molecular Pathology, National Institute of Oncology, Budapest, Hungary; c Department of Genitourinary Medical Oncology and Clinical Pharmacology, National Institute of Oncology, Budapest, Hungary; d Department of Molecular Genetics, National Institute of Oncology, Budapest, Hungary; e HUN-REN Hereditary Cancer Research Group, Budapest, Hungary; f Central Biobank, National Institute of Oncology, Budapest, Hungary; g Center of Radiotherapy, National Institute of Oncology, Budapest, Hungary; h Center of Radiotherapy, National Institute of Oncology, Budapest, Hungary; i Department of Molecular Immunology and Toxicology, National Institute of Oncology, Budapest, Hungary; j Center of Radiotherapy, National Institute of Oncology, Budapest, Hungary

**Keywords:** Tumour mutation profile, molecular tumour board, targeted therapy

## Abstract

Recent developments in molecular genetic testing methods (e.g. next-generation sequencing [NGS]-panels) largely accelerated the process of finding the most appropriate targeted therapeutic intervention for cancer patients based on molecularly targetable genetic alterations. In Hungary, a centralized approval system following the recommendation of the National Molecular Tumor Board was launched for the coordination of all aspects of comprehensive genetic profiling (CGP) including patient selection and therapy reimbursement.

**Aim:**

The study aims to evaluate the clinical benefit of CGP in our Comprehensive Cancer Center

**Methods and patients:**

CGP was introduced into our routine clinical practice in 2021. An NGS-based large (> 500 genes) gene panel was used for cases where molecular genetic testing was approved by the National Molecular Tumor Board. From 2021 until August 2023 163 cases were tested. The majority of them were ECOG 0–1 patients with advanced-stage diseases, histologically rare cancer, or cancers with unknown primary tumours.

**Results:**

Seventy-four cases (74 of 163, 45%) had clinically relevant genetic alterations. In 34 patients, the identified variants represented an indication for an approved therapy (approved by the Hungarian authorities, on-label indication), while in 40 cases the recommended therapy did not have an approved indication in Hungary for certain tumour types, but off-label indication could be recommended. Based on our CGP results, 24 patients (24/163; 14.7%) received targeted therapy. Treatment duration was between 1 and 60 months. In total 14 (14/163; 8.5% of the tested cases) patients had a positive clinical response (objective response or stable disease) and were treated for more than 16 weeks.

**Interpretation:**

NGS-based CGP was successfully introduced in our institution and a significant number of patients benefited from comprehensive genetic tests. Our preliminary results can serve as the starting point of Drug Rediscovery Protocol (DRUP) studies.

## Background

In routine clinical practice various molecular genetic tests are used for the identification of therapeutically actionable genetic variants. Single gene tests, various-sized gene panels, or large-scale genetic analyses evaluating > 500 genes, whole exome, and whole genome sequencing are available. In our routine clinical practice, the next-generation sequencing (NGS) approach that uses a single assay to assess approximately 500 genes and genetic variants including relevant cancer biomarkers, as established in guidelines and clinical trials, for therapy guidance in cancer patients has been introduced in 2021 [1]. We refer to this analysis as ‘Comprehensive genomic profiling-CGP’.

The availability of NGS-based comprehensive genetic profiling (CGP) in Europe is not uniform. Some countries have already introduced its use in routine clinical practice while in others it is not utilized. Limited access to molecular pathology, clinical genetics, and genomics expertise are among the reasons behind the latter cases, representing considerable inequalities in oncology care in Europe.

CGP offers important benefits to identify molecular alterations that can be used as a therapeutic target [2]. Using it in routine clinical practice is challenging due to the associated high costs and required expertise [3]. In addition to specialists in molecular genetic diagnostics, molecular pathology, clinical genetics, oncology, and bioinformatics, significant infrastructural investments are also needed. The benefit and cost-effectiveness of CGP over smaller targeted gene panels have not yet been unambiguously demonstrated. Indications for CGP, according to the The European Society for Medical Oncology (ESMO)guideline, include patients with rare tumours, cancers with unknown primary (CUP), when the therapeutic options have been exhausted but the patient is still in good condition and tumours where the therapeutic indication is based on genomic instability score (GIS) or high tumour mutational burden [4].

To test the clinical benefit of CGP in Hungary the National Health Insurance Fund of Hungary (Hungarian acronym: NEAK) initiated a nationwide molecular tumour board (MTB).

NEAK is the central agency that manages the National Health Insurance Fund and as the only health-related funding agency of the government in Hungary it reimburses all expenses [5]. At four university centers and the National Institute of Oncology various molecular genetic testing methods were introduced into the clinical practice. Comprehensive, large gene panel testing is available from 2019 at two centers in the National Institute of Oncology and Semmelweis University, and from 2022 at the University of Pécs.

MTBs are heterogeneous and a various-sized group of healthcare professionals whose expertise guarantees the most effective workflow and recommendations for cancer patients regarding therapeutic decisions [6]. There are different MTBs: usually every oncology centre where molecular genetic tests are performed has an MTB. However, their size, their members, and their workload can be significantly different. Some are involved at specialized centers (i.e. oncohematology, pediatric cancer etc.) while others (i.e. large centers, typically working closely with comprehensive cancer centers) cover multiple cancer types [7].

Our current work summarizes the steps of the Hungarian Precision Cancer Medicine project started in 2019 at the National Institute of Oncology, Comprehensive Cancer Center, Budapest supported by the Hungarian authorities in line with two European Union-funded projects: PCM4EU (Personalized Cancer Medicine for all EU citizens) and PRIME-ROSE (Precision Cancer Medicine Repurposing System Using Pragmatic Clinical Trials) aim to improve the implementation of molecular genetic test results in direct patient care.

## Methods

### Initiation of the national molecular genetics and rare cancer tumor board

In our institute, NGS-based assays, mainly smaller targeted panels, were implemented in molecular pathology diagnostics in 2019, and parallel, genetic counseling with comprehensive germline genetic testing for cancer patients has been introduced into routine clinical workflow.

Ordering and availability of molecular genetic testing were performed according to the Hungarian law in a bespoke testing pathway. All patients’ samples were reviewed by a local pathologist and the type of molecular test was determined by the tumour cell content and size of the sample in addition to the clinical and pathological diagnosis. In some cases, additional immunohistochemical test was performed by our pathologists to confirm the external diagnosis before initiating the molecular tests.

From the end of 2019 the Hungarian Government and Health Insurance Office (NEAK), to help provide nationwide availability to comprehensive molecular genetic tests, approved the formation of the Molecular Genetics and Rare Cancer Tumor Board, referred to as the National Molecular Tumor Board (NMTB). Requests for NGS-based molecular genetic testing are open for all oncological centers and for all cancer patients from the country. The Hungarian NMTB consists of multi-disciplinary and interdisciplinary expert panel members including four pathologists (two experts in molecular pathology and molecular genetic diagnostics), three physicians specialized in clinical and molecular genetic diagnostics, and clinical genetics, four clinical oncologists, two physicians specialized in radiotherapy, one molecular biologist expert in oncohematology. This board is accompanied by one representative of the NEAK who participates in the weekly meetings. The NMTB reviews the anonymous documentation of patients for whom genetic testing is requested. On average 10–15 cases per week are evaluated. Patients should be at clinical status ECOG 0 or 1. The NEAK provides all relevant documentation to the members of NMTB for evaluation through a secure online platform at least 24 h before the meeting. All previous pathology reports (including results obtained with smaller gene panels), clinical data, and previous therapies are reviewed. During the NMTB meeting, a consensus recommendation is issued and sent back to NEAK who transfer this to the treating physician.

The NGS-based molecular genetic testing is reimbursed for cancer patients by the Health Insurance Office. CGP for somatic testing was introduced in 2021 in our institute. Between December 2021 and August 2023, based on the recommendation of the NMTB 163 cases were tested using CGP. All patients have given consent for participating in the study.

### Patients and comprehensive molecular genetic profiling

Of the 163 samples, 109 were primary and 54 were metastases. The distribution of sample types was as follows: 1 cell block, 2 cytology smears, 47 biopsy samples, and 113 resection specimens. Blocks were not older than 3 years.

The localization of tumours is presented in Table 1. Soft tissue, urogenital tumours including high-grade tubo-ovarian serous carcinoma (HGSOC) and cancers of the gastrointestinal system (including pancreatic cancers) were the most prevalent tumour types tested. The common tumours including that is breast carcinoma or lung carcinomas are routinely evaluated by smaller NGS gene panels; therefore, these types are underrepresented in this analysis.

**Table 1:** Localization of tumours and the number of patients tested by comprehensive genetic profiling.

Tumour type and localisation	Number of cases
Breast cancer	3
CUP (cancer with unknown primary)	6
Gastrointestinal tumours (including pancreatic cancer, *n* = 4)	23
Head and neck cancer	6
Lung cancer	7
Soft tissue tumour	39
Thyroid cancer	5
Prostate cancer	5
High-grade serous ovarian cancer (HGSOC)	16
Other tumors of the urogenital system	30
Other tumor types	23

Tumour DNA and RNA were extracted from formalin-fixed paraffin-embedded (FFPE) tissue blocks. Hematoxylin-eosin (HE)-stained sections of all samples were reviewed by a pathologist to estimate the tumour cell content and select the tumorous part for macrodissection. Nucleic acid isolation was performed using either Maxwell RSC DNA/RNA FFPE Kit on Maxwell RSC Instrument (Promega, USA) or MagMAX FFPE DNA/RNA Ultra Kit on the KingFisher Duo Prime purification system (Thermo Fisher Scientific, USA) according to the manufacturer’s instruction. DNA and RNA concentrations were measured using a Qubit Fluorometer with Qubit dsDNA HS Assay and Qubit RNA HS Assay Kit (Thermo Fisher Scientific). Before sequencing the quality of DNA samples was determined using the TaqMan RNase P Detection Reagents Kit (Thermo Fisher Scientific) by quantifying the presence of amplifiable DNA molecules. Samples with ΔCt ≤ 2 compared to the control, predicted their suitability for NGS. QC parameters and tumour cell contents are available on request. The average of tumour cell content was 59% and there were 17 cases with less than 30% tumour cell content.

DNA and RNA libraries were prepared separately with 20–40 ng of input amount and constructed by automated library preparation using the Ion Chef Instrument and Oncomine Comprehensive Assay Plus kit (Thermo Fisher Scientific). Sequencing was performed on the Ion S5 Plus Sequencer. Parameters used for assessing run quality included key signal > 100, Ion Sphere Particles (ISP) loading > 85%, and usable reads > 40%. Parameters used for assessing DNA sample quality included mean read depth >800×, Median Absolute Pair-wise Difference (MAPD) (for copy number variation [CNV] calling)< 0.5, deamination score (for tumor mutation burden [TMB] determination) < 20, and uniformity > 90%. RNA quality metrics included total valid mapped reads > 500,000×, and mean read length > 40 base pairs.

Sequencing data were analyzed using the Torrent Suite Software and Ion Reporter Software on the Torrent Server for automated sequencing data alignment and analysis. Base calling, alignments, and run quality control were performed using the Torrent Suite™ Software v5.18.1. Variant calling, annotation, and assessing TMB, microsatellite instability score (MSI), and homologous recombination (HRD) with the GIS were calculated by Ion Reporter Software 5.20 Workflow Version:3.1.

To make a CNV call the following criteria must be met: MAPD < 0.4, CNV ratio for a copy number gain must be > 2, *P* < 10^-5^, CNV ratio for a copy number loss must be < 0.85.

A sample-level MSI score is calculated with 76 individual MSI marker’s scores. The overall score is used to determine the MSI status of the sample. In case of MSI-H tumors this score is ≥ 18.

The genomic instability metric (GIM) or genomic instability status (GIS) is the same as HRD score. It is a numeric value between 0 and 100 that summarizes unbalanced copy number changes that comes from loss of heterozygosity (LOH), large-scale transitions (LST), and telomeric allelic imbalance (TAI) using genomic segmentation analysis. Higher GIM values correlate with the observation of more genomic instability in the sample. The cutoff value was set for patients with high-grade serous ovarian carcinoma validated on clinical data.

We used the The American College of Medical Genetics and Genomics classification system for variant interpretation by applying online databases (Clinvar, Varsome, Franklin).

## Results

In 152 of 163 cases, all QC parameters were appropriate for performing the analysis. In 6 of 163 cases detection of fusion transcripts failed and in 6 cases the determination of TMB failed. The determination of the LST and telomeric allelic imbalance (TAI) indices and GIS could not be determined in eight samples.

Out of the 163 cases tested, 74 cases (45%) had actionable genetic variants. In 34 patients, the identified variants represented an indication for an approved therapy (on-label group), while 40 cases represented an off-label indication in Hungary for the actual tumor type. Off-label indication in our practice means that there is an available The Food and Drug Administration (FDA) and European Medicines Agency (EMA)-approved drug for certain genetic alteration but due to national decisions for certain tumours types is not reimbursed in Hungary, and additionally, the ESCAT level III-IV therapies are also included in this group. In these cases, individual permission approved by the NEAK was required to start the therapy.

**Figure 1:**
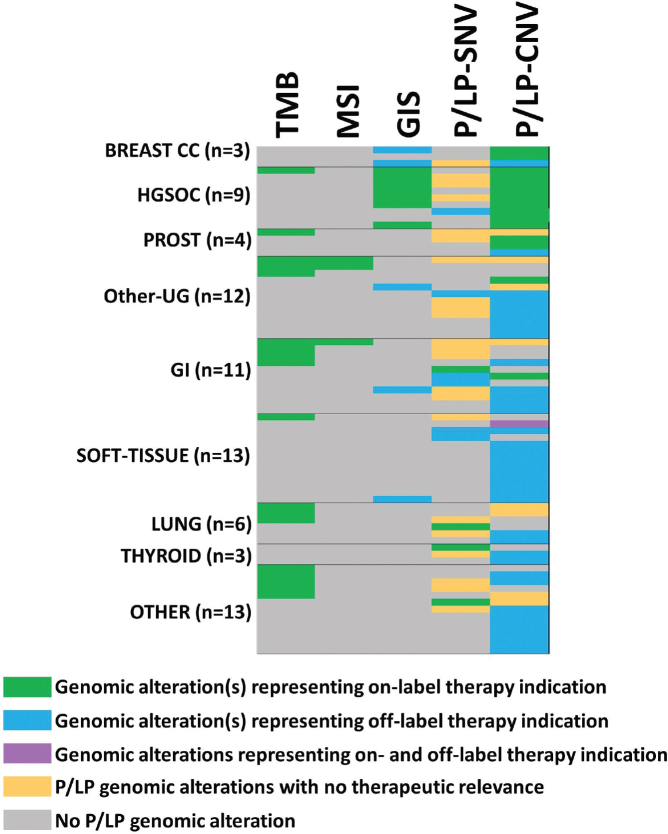
Genetic alterations representing therapeutical indication identified using CPG. Green labels indicate the main P/LP genetic alteration representing an indication for on-label therapy (there can be more than one genetic alteration representing an indication for treatment in the same person). Blue color used for P/LP genetic alteration represents an indication for off-label therapy. Orange color is used for P/LP genetic alteration representing no therapy indication while grey color shows cases without any P/LP variants. The lilac color indicated that there were two genetic alterations; one with on-label (CDK4 CNV), and one with off-label (BRCA2 CNV) indication. P/LP: pathogenic and likely pathogenic variants called by ACMG criteria. CNV: copy number variation.

The distribution of genetic alterations representing therapeutical indications is summarized in Figure 1. High TMB followed by copy number alterations of *BRCA1/2* genes were the most common findings which indicate on-label therapeutical indication (Table 2).

**Table 2:** Genetic alterations associated with high TMB in tumours tested by comprehensive genetic profiling.

Tumour type	Pathogenic variants SNV	LOH	Amplification	Deletion, duplication	Fusion	TMB (Mutations/Mb)
High-grade serous ovarian cancer (HGSOC)	n.d.	BRCA1, BRIP1, CDK12, PALB2, POLD1, POLE, PTEN, RAD51B, RAD51C, RAD51D, RAD54L	n.d.	n.d.	Negative	10.52
Brain metastasis of prostate adenocarcinoma	PALB2 exon 4, p.Met296Ter, c.886delA	PPP2R2A, PTEN	n.d.	n.d.	TMPRSS2 – ERG	11.39
Endometrial adenocarcinoma	MSH6 exon 7: c.35571G > C, APC exon 7: p.Arg232Ter, c.694C > T, ATM exon 35: p.Arg1730Ter, c.5188C > T, PTEN exon 5: p.Arg130Ter, c.388C > T, PTEN exon 7: p.Pro246Leu, c.737C > T, ERBB2 exon 17: p.Arg678Gln, c.2033G > A	n.d.	MYC	HLA-A, HLA-B, CDKN2A, ERAP2	Negative	31.41
Endometrial carcinoma metastasis	n.d.	n.d.	n.d.	del: CDKN2A, HLA-B, HLA-A, ERAP2	Negative	35.76
Clear cell ovarian carcinoma	n.d.	n.d.	n.d.	n.d.	Negative	24.82
Adenocarcinoma of the transverse colon	KRAS exon 4: p.Ala146Thr, c.436G > A	n.d.	n.d.	n.d.	Negative	18.97
Colorectal adenocarcinoma	APC exon 16, p.Tyr935Ter, c.2805C > A	n.d.	n.d.	del: NCOR1, CDKN2A, ERAP2	Negative	44.38
Colorectal adenocarcinoma	POLE exon 9: p.Pro286Arg, c.857C > G	n.d.	n.d.	del: CDKN2A	Negative	112.07
Oesophageal adenocarcinoma	not detected	BRCA2 LOH 13q13		BRCA1 exon2–18 duplication	Negative	13.29
Cutaneous angiosarcoma	HRAS exon 3: p.Gln61Leu, c.182A > T			del: CDKN2A, HLA-B	Negative	12.43
Lung adenocarcinoma	not detected	POLD1	MET, FAM135B ; MYC	n.d.	Failed	13.32
Lung large cell neuroendocrine carcinoma	not detected	CHEK2, NBN, POLD1, PPP2R2A, PTEN	MYC	n.d.	Negative	21.01
Lung poorly differentiated carcinoma with neuroendocrine differentiation	LP POLE exon42: p.Gly1923Cys, c.5767G > T,	n.d.	n.d.	n.d.	Negative	32.8
Parathyroid carcinoma	n.d.	n.d.	n.d.	del: CDKN2A, HLA-A, ERAP2	Negative	10.43
High-grade neuroendocrine carcinoma lymph node metastasis	n.d.	BRCA2, BARD1, BLM, CHEK2, FANCL, PALB2, PTEN, RAD51B,	KIT, PDGFRA	n.d.	Negative	16.16
Retroperitoneal high-grade neuroendocrine carcinoma	ATM exon 43: p.Tyr2100Ter, c.6300C > A,	BRCA2	MDM2; DDR2, NFE2L2	n.d.	Negative	87.21
Melanoma metastasis	NRAS exon 3: p.Gln61Leu, c.182A > T,	n.d.	n.d.	n.d.	Negative	39.62
Glioblastoma	n.d.	n.d.	EGFR, PDGFRA	del: PDIA3, MGA, RAD51, TCF7L2, SUFU, CYP2C9, PTEN, ARID5B, MAPK8, GATA3, LARP4B, CDKN2B, CDKN2A, MTAP, HLA-B, HLA-A, EPHA2, SPEN, PGD,	Negative	10.46

n.d.: not detected; del: deletion; TMB: tumor mutation burden.

Regarding tumour types, the highest percentage of genetic alterations with therapeutical targets were identified in breast, lung and prostate carcinomas; however, in these tumour types low number of cases were evaluated. Immunotherapy is approved in Hungary based on the TMB score besides PD-L1 expression. PARP inhibitor therapy is approved for HGSOC, prostate and pancreatic cancer based on alterations of *BRCA1/2* genes or HRD index (Table 3).

**Table 3:** Number and percentage of cases by tumour type harboring therapeutically actionable genetic alterations identified by comprehensive genetic profiling.

Tumor type and localisation	Number of cases tested	Number of cases with targetable genetic alterations	Percentage of targetable genetic alterations (%)
Breast cancer	3	3	100
CUP (cancer with unknown primary)	6	0	0
Gastrointestinal tumours	23	11	48
Head and neck cc.	6	0	0
Lung carcinoma	7	6	85
Soft tissue sarconoma	39	13	33
Thyroid cc.	5	3	60
Prostate cc.	5	4	80
High-grade serous ovarian cancer (HGSOC)	16	9	56
Other tumours of the urogenital system	30	12	40
Other tumour types	23	13	56
Total	163	74	45

NGS-based tests containing a smaller number of genes are also routinely used in our institute for cancer types where approved therapies rely on genetic alterations covered by these panels (such as breast, HGSOC, lung, prostate cancer, colon, and endometrial cancer). During the same period, 1338 smaller targeted gene panel tests and 346 somatic *BRCA1/2* tests were performed based on the local decision of molecular pathologists, pathologists, and clinical oncologists. These decisions are made within the organ-specific oncoteams routinely performed at our institute. Using the two smaller targeted panels, of 1338 tested cases 488 (36.5%) cases had a genetic variant that could represent an indication for targeted therapy. In 422 (31.5%) cases the ESCAT (ESMO Scale for Clinical Actionability of Molecular Targets) level I, in 17 cases ESCAT level II, in 43 cases ESCAT level III, and in 6 cases ESCAT level IV therapeutic indications were identified. Of the 346 cases, somatic pathogenic mutations in one of the *BRCA1/2* genes were identified in 97 cases, which represents 28% of all tumours tested, predominantly HGSOC, breast and prostate cancer.

Regarding therapeutical decisions based on CGP results, in total, 24 patients (24/163; 14.7%;11 out of 34 patients received on-label and 13 out of 40 patients received off-label therapy) received therapy (Table 4). The treatment duration was between 1 and 60 months. In total 14 (14/163; 8.5% of the tested cases) patients had a positive clinical response (objective response or stable disease) and were treated for more than 4 months (16 weeks). This is consistent with previously reported data [8, 9]. Treatments were stopped due to toxicity or disease progression.

**Table 4:** Therapeutical intervention based on the genetic alterations identified with comprehensive genetic profiling.

Tumour type	Genetic alterations	TMB status	Therapy	Treatment duration (month) and reason of termination	
**On-label therapy**					
Metastasis of HGSOC	BRCA1 p.Glu23ValfsTer17, c.68_69delAG,^X^	low	PARP inhibitor	14	Progression
Colon carcinoma	KRAS p.Ala146Thr, c.436G > A	high^X^	Immunotherapy	60	On therapy
Metastasis of HGSOC	BRCA1 p.Gln1604AsnfsTer2, c.4806delT^X^ and LOH of BRCA1, BRCA2, BARD1, BRIP1, POLD1, POLE	low	PARP inhibitor	6	Progression
HGSOC	LOH: BRCA1^X^, BRIP1, CDK12, PALB2, POLD1, POLE, PTEN, RAD51B, RAD51C, RAD51D, RAD54L	failed	PARP inhibitor	5	Toxicity
Lung carcinoma	MET amplification^X^ and LOH of POLD1	high^X^	MET TKI (Crizotinib) and immunotherapy	4	Progression
Large cell lung neuroendocrine carcinoma	LOH: CHEK2, NBN, POLD1, PPP2R2A, PTEN	high ^X^	Immunotherapy	8	On therapy
Breast carcinoma	Amplification of BRCA2 exons: 2–11^X^	low	PARP inhibitor	4	On therapy
Metastasis of melanoma	Deletion of BRCA2 exons 15–16 and exons 19–20; NRAS exon 3: p.Gln61Leu, c.182A > T,	high^X^	Immunotherapy	4	Toxicity
Ovarian adenocarcinoma	LOH: BRCA1 ^X^, BRIP1, CDK12, PALB2, POLD1, RAD51C, RAD51D, RAD54L	low	PARP inhibitor	5	On therapy
Rectum carcinoma	APC p.Tyr935Ter, c.2805C > A and del NCOR1, CDKN2A, ERAP2	high^X^	Immunotherapy	1	Death
Metastasis of endometrial carcinoma	MSH6 exon 9, p.Arg1331Ter, c.3991C > T^X^, deletions: HLA-B, HLA-A, ERAP2	low	Immunotherapy	1	Death
**Off-label therapy**					
Cholangiocarcinoma	LOH: BRCA2^X^, POLE, PPP2R2A	low	PARP inhibitor	11	Progression
Metastasis of breast cancer	dup: BRCA1^X^, BRCA2^X^ and LOH: ATM, CHEK1, PPP2R2A, RAD51B	low	PARP inhibitor	1	Toxicity
Small cell lung cancer	LOH: BRCA2^X^, CHEK2, PTEN, RAD54L; BRCA1, BRCA2	low	PARP inhibitor	4	Progression
Metastasis of thymoma	LOH: RAD51B^X^	low	PARP inhibitor	4	On therapy
High-grade sarcoma	LOH: BRCA1,^X^ BRCA2, ATM, BARD1, CHEK1, CHEK2, PPP2R2A, RAD51B	low	PARP inhibitor	2	Progression
Malignant peripheral nervous sheet tumor (MPNST)	deletion BRCA2 exons 10–27^X^	low	PARP inhibitor	5	Progression
Testicular embrional carcinoma	amplification MET^X^	low	cabozantinib	6	Progression
Parathyroid carcinoma	deletion: CDKN2A, HLA-A, ERAP2	high^X^	immunotherapy	11	On therapy
Leiomyosarcoma (rectosigma)	LOH: BRCA2^X^, ATM, CHEK1, CHEK2, POLE, PPP2R2A	low	PARP inhibitor	8	Progression
High-grade spindle cell sarcoma	LOH: BRCA1^X^, BRCA2, ATM, BRIP1, CDK12, CHEK1, NBN, PALB2, POLE, PPP2R2A, PTEN, RAD51B, RAD51C, RAD51D	low	PARP inhibitor	1	Progression
Metastatic germcell tumor	LOH: BRCA1^X^, ATM, BARD1, BLM, CHEK1, FANCL	low	PARP inhibitor	6	On therapy
Metastasis of leiomyosarcoma	deletion BRCA2 exon 16–20 ^X^	low	PARP inhibitor	1	Toxicity
Metastasis of postpubertal teratoma	LOH: BARD1^X^, CHEK2	low	PARP inhibitor	2	Progression

TMB: tumor mutation burden;

X : indicates the genetic alteration representing an indication for therapy.

Reasons for not receiving the recommended targeted therapy in both groups were rapid disease progression, death, or the unavailability of the suggested therapy.

## Discussion

Despite the relatively short period of the study, the identified proportion of patients with actionable genomic alterations and the ones who received therapy based on the CGP result, as well as the response and the disease control rates were consistent with previously published data [10].

CGP is a valid and important method for the identification of cases with potentially targetable genetic alterations. This is particularly important for patients where the therapeutical options are limited or the identification of the tumour type is challenging (cases with rare cancers or unknown primary).

In addition to CGP, our centre still uses other, smaller gene panels for routine diagnostic testing mostly because of operational considerations including optimal laboratory workflow, sample size, low tumour cell content frequently detected in certain tumour types, shorter turnaround time of the results, and costs. In addition, in many common cancers, all targetable genetic variants are identifiable by these validated and certified assays, and in these cases, only the TMB and GIS evaluation require CGP [11]. For this reason, the number of common tumours with multiple possible targeted therapies was relatively low in our study. In addition, patients who benefit from therapies like: MEK-inhibitors, BRAF-inhibitors, PI3K-inhibitors, and CDK4/6-inhibitors are often identified using smaller gene panels; therefore, these cases are missing from this cohort (data not shown).

A significant proportion (two-thirds) of the samples tested were primary tumors, with rare histology types where no targeted therapies are available. These cases were tested before or during the first progression. The third part tested were metastases and these patients had several lines of therapy. In this group, any potential actionable genetic variants are very important. Our data showed that nine cases tested from metastases received targeted therapy, and half of them had good clinical response (Table 4).

The high proportion of cases showing actionable targets but not having approved therapy represents a constant challenge. Our data underline the need for a rapid decision from the financial body or, where it is not available, from industrial partners to start the recommended therapy as soon as possible. Finding an adequate ongoing clinical trial was not achievable for our cases either due to the late disease stage or limited ongoing trials.

From January 2023 the European Union financed ‘Personalized Cancer Medicine for European Citizens’ (PCM4EU) project was launched. Our Institute represents Hungary in this project. Our current work is consistent with the main goals of the PCM4EU project (PCM4EU website (pcm4eu.eu). Having in our disposition the complex molecular genetic workflow including CGP along with complex germline testing we can identify patients in whom a Drug Rediscovery Protocol (DRUP) could be initiated [12]. The DRUP-like trials are prospective phase II combined umbrella-basket trials. The selection of patients in these trials is based on the genomic alterations present in their tumours. Patients with advanced cancers receive targeted therapies based on tumour type and molecular alterations relevant to targeted therapy.

Our report is one of the first reports showing a national effort to introduce CGP into clinical practice. The Hungarian health system and its openness to innovation are unique among European countries. Our practice together with DRUP trials provided firm justification for reimbursement of treatments, which are indicated by CGP results [13, 14].

In summary, our NMTB, established in December 2019 is unique in Europe or worldwide because it is coordinated by the health insurance provider of the country. All decisions are based on experts’ opinions and the results and recommendations are immediately translated into clinical practice.

## Data Availability

All data are included in the manuscript. Further queries can be directed to the corresponding author.
